# Artificial intelligence in healthcare: transforming patient safety with intelligent systems—A systematic review

**DOI:** 10.3389/fmed.2024.1522554

**Published:** 2025-01-08

**Authors:** Francesco De Micco, Gianmarco Di Palma, Davide Ferorelli, Anna De Benedictis, Luca Tomassini, Vittoradolfo Tambone, Mariano Cingolani, Roberto Scendoni

**Affiliations:** ^1^Research Unit of Bioethics and Humanities, Department of Medicine and Surgery, Università Campus Bio-Medico di Roma, Rome, Italy; ^2^Department of Clinical Affair, Fondazione Policlinico Universitario Campus Bio-Medico, Rome, Italy; ^3^Department of Public Health, Experimental and Forensic Sciences, University of Pavia, Pavia, Italy; ^4^Interdisciplinary Department of Medicine (DIM), Section of Legal Medicine, University of Bari “Aldo Moro”, Bari, Italy; ^5^Research Unit of Nursing Science, Department of Medicine and Surgery, Università Campus Bio-Medico di Roma, Rome, Italy; ^6^International School of Advanced Studies, University of Camerino, Camerino, Italy; ^7^Department of Law, Institute of Legal Medicine, University of Macerata, Macerata, Italy; ^8^Italian Network for Safety in Healthcare (INSH), Coordination of Marche Region, Macerata, Italy

**Keywords:** artificial intelligence, patient safety, healthcare, intelligent systems, machine learning

## Abstract

**Introduction:**

Adverse events in hospitals significantly compromise patient safety and trust in healthcare systems, with medical errors being a leading cause of death globally. Despite efforts to reduce these errors, reporting remains low, and effective system changes are rare. This systematic review explores the potential of artificial intelligence (AI) in clinical risk management.

**Methods:**

The systematic review was conducted using the PRISMA Statement 2020 guidelines to ensure a comprehensive and transparent approach. We utilized the online tool Rayyan for efficient screening and selection of relevant studies from three different online bibliographic.

**Results:**

AI systems, including machine learning and natural language processing, show promise in detecting adverse events, predicting medication errors, assessing fall risks, and preventing pressure injuries. Studies reveal that AI can improve incident reporting accuracy, identify high-risk incidents, and automate classification processes. However, challenges such as socio-technical issues, implementation barriers, and the need for standardization persist.

**Discussion:**

The review highlights the effectiveness of AI in various applications but underscores the necessity for further research to ensure safe and consistent integration into clinical practices. Future directions involve refining AI tools through continuous feedback and addressing regulatory standards to enhance patient safety and care quality.

## 1 Introduction

Adverse events in hospitals pose a serious threat to patient care quality and safety globally, contributing to patient distrust and impacting healthcare facility reputations (1). A significant report estimated 45,000–98,000 annual deaths in the U.S. due to medical errors (2). Despite widespread reporting systems, < 10% of errors are reported, and only 15% of hospital responses prevent future incidents (3). Overcoming structural and cultural barriers is crucial for improving patient safety (4). Medical errors, defined as actions leading to unintended results, affect patients, families, healthcare providers, and communities (5). They include drug side effects, misdiagnoses, surgical errors, and falls (7), occurring across care processes from medication to post-operative care. Healthcare risk management combines reactive systems like incident reporting with proactive methods such as Failure Mode and Effects Analysis (FMECA) (8), aiming to learn from past errors and prevent future ones through continuous improvement. Artificial Intelligence (AI) offers potential in healthcare by enhancing diagnostics, optimizing care, and predicting outcomes (9, 10). AI can detect clinical data anomalies, improving diagnostic accuracy, though integrating AI requires addressing new and existing risks (11, 12). This review provides an overview of AI applications in clinical risk management, assessing their benefits, reproducibility, and integration challenges in healthcare settings.

## 2 Materials and methods

The methodology of this systematic review was developed following the guidelines of The Preferred Reporting Items for a Systematic Review and Meta-Analysis of Diagnostic Test Accuracy Studies (PRISMA-DTA) (13).

### 2.1 Keywords Identification

The keywords for the search (Table 1) were selected using terms related to the phrases “clinical risk management” and “artificial intelligence.” The search string used is provided in Table 1.

**Table 1:** Serch string.

**Search string**
((“risk management”) OR (“clinical governance”) OR (risk assessment) OR (risk prediction)) AND ((“patient safety”) OR (“safety in healthcare”) OR (“quality in healthcare”)) AND ((artificial intelligence) OR (machine learning) OR (deep learning) OR (artificial neural networks))

### 2.2 Search strategy

The search of the scientific literature was conducted in February 2024. Three online bibliographic databases were examined, which are as follows:

- Pubmed
- Scopus
- Web of Science

The first phase of the literature review was carried out using the Rayyan^®^ tool.

### 2.3 Inclusion and exclusion criteria

This systematic review includes studies that simultaneously meet all of the following criteria: (1) Use of artificial intelligence systems, defined as any system capable of replicating complex mental processes through the use of a computer. (2)Application of the artificial intelligence system in the healthcare context. (3) Employment of the artificial intelligence system in areas of interest to clinical risk management. (4) Presence of results derived from the active experimentation of the system. (5) Prevention of an adverse event, defined as an unintentional incident resulting in harm to the patient's health that is not directly related to the natural progression of the patient's disease or health condition (14).

Exclusion criteria were primarily used to remove studies that, although involving the use of artificial intelligence systems to enhance care safety, addressed areas not pertinent to the concept of medical error (e.g., risk of cardiac arrest, risk of re-infarction, etc.).

## 3 Results

**Figure 1:**
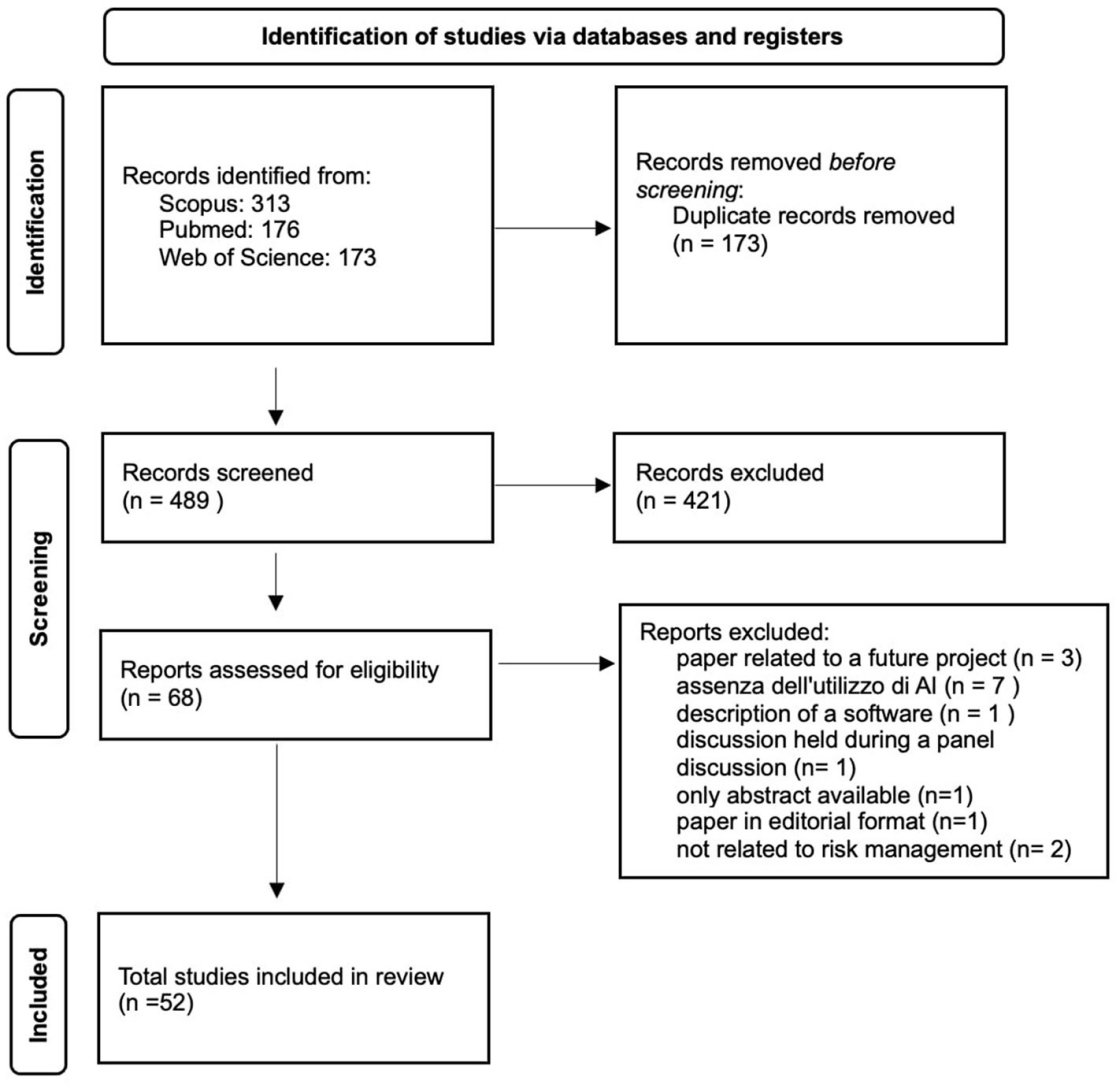
Identification of studies via databases and registers.

The search across the three databases yielded 662 results (Figure 1). After removing duplicates, the number was reduced to 489 studies. We excluded 421 articles as they did not meet the five established inclusion criteria. In most cases, the excluded events pertained to contexts unrelated to clinical risk management, such as complications arising from the natural progression of diseases rather than preventable adverse events. Following an initial review of the articles, 68 studies were included in the database. An additional 16 studies were excluded. One of the articles (15) was excluded because it represents a future development project for a high-performance prediction, detection, and monitoring platform for managing risks against patient safety, without providing any results. Seven of the articles (16–22) were not included as they addressed clinical risk topics but did not reference the use of artificial intelligence. One of the excluded articles (23) was only available as an abstract. Three studies were not included because, although they discussed the use of artificial intelligence in clinical risk management, they only described the software without reporting results (22, 24, 25). One article was excluded as it was a report of a discussion from a roundtable on risk management in the use of medical devices (26). Another article was not included because it was an editorial and did not meet the inclusion criteria (27). Two articles were excluded as they were not relevant to risk management in the hospital environment (28, 29).

### 3.1 Analysis and findings

The analyzed studies propose diverse methodologies in the field of risk management, offering both proactive and reactive approaches within a heterogeneous application context. The main characteristics of the reviewed articles are presented in Table 2.

**Table 2:** Main objective and type of approach to risk of reviewed publications.

**References**	**Year**	**Risk approach**	**Main objective**
(67)	2023	Proactive	The study reviews and evaluates research on machine learning prediction models to identify pressure injury risks in adult hospitalized patients.
(30)	2021	Reactive	The study proposes a method to compute adverse events underreporting probability, enhancing patient safety and reducing manual QA in clinical trials.
(31)	2021	Proactive and reactive	This review evaluates AI's potential to enhance patient safety in domains such as infections, adverse drug events, thromboembolism, surgical complications, pressure ulcers, falls, decompensation, and diagnostic errors.
(32)	2016	Reactive	The aim is to develop a reliable electronic approach for processing text in medical event reports to enhance patient safety.
(68)	2007	Proactive	The aim is to develop a reproducible approach integrating human qualitative coding patterns with machine learning.
(33)	2022	Proactive	The aim is to create a reproducible method for integrating human qualitative coding patterns with machine learning.
(34)	2024	Reactive	The study aims to investigate the effectiveness of machine learning classifiers trained with contextual text representations in automatically classifying patient safety event (PSE) reports.
(57)	2021	Proactive	The purpose of the study is to determine the impact of an electronic analytic tool for predicting fall risk on patient outcomes and nurses responses.
(58)	2023	Proactive	The aim is to assess whether a fall-prevention clinical decision support approach using electronic analytics that stimulates risk-targeted interventions is associated with reduced rates of falls and injurious falls.
(79)	2020	Proactive and reactive	The objective of this review is to identify and analyze quantitative studies utilizing or integrating artificial intelligence to address and report clinical-level patient safety outcomes.
(53)	2020	Proactive	The main objective is to improve patient safety and clinical outcomes by reducing the risk of prescribing errors, we tested the accuracy of a hybrid clinical decision support system in prioritizing prescription checks.
(69)	2022	Proactive	To analyze pressure injury risk factors, to identify strong predictors and to use different machine learning algorithms to classify patients with pressure injury and patients without pressure injury.
(35)	2015	Reactive	The study proposes a method to automatically classify/label event reports via semi-supervised learning which utilizes labeled as well as unlabeled event reports to complete the classification task. It focuses on classifying two types of event reports: patient mismatches and weight errors.
(36)	2017	Reactive	The aim of the paper is to develop a more efficient and streamlined method for categorizing patient safety event reports based on modeling the free text of event reports to reduce the review time of the committee.
(37)	2012	Reactive	The aim is to accumulate and reinterpret findings using structured incident information, to clarify improvements that should be made to solve the root cause of the accident, and to ensure safe medical treatment through such improvements
(38)	2013	Reactive	The aim of the study is the development of a method based on natural language processing to quickly search electronic health records for common triggers and adverse events.
(39)	2015	Reactive	The study compares the performance of different machine learning classifiers on a dataset of documents labeled by clinicians and experts.
(40)	2019	Reactive	In this study C4.5 decision tree, a single classifier, and Random Forest (RF), an ensemble classifier, are investigated to train and validate three multiclass Clinical Safety Incident taxonomies.
(41)	2021	Proactive	The aim of this study was to explore whether employing text mining techniques on patient complaint databases can help identify potential problems with patient safety at health care providers and automatically predict the severity of patient complaints.
(59)	2022	Proactive	In the study a machine learning technique is used to analyze events involving falling and establish a risk prediction model
(78)	2023	Proactive	In the study an Artificial Intelligence Clinical Assistant Decision Support System was used for venous thromboembolism prophylaxis of inpatients.
(54)	2021	Proactive	The paper proposes the use of machine learning approaches for characterizing the risk factors associated with medication ordering errors. Toward this end, we evaluated the performance of multiple machine learning methods on a large dataset of self-intercepted medication ordering errors.
(60)	2022	Proactive	The aims of this study were to create a model that detects the population at risk of falls taking into account a fall prevention variable and to know the effect on the model's performance when not considering it.
(70)	2020	Proactive	The aim of the study is to build a model to detect pressure injury risk in intensive care unit patients and to put the model into production in a real environment.
(61)	2021	Proactive	The purpose of this study was to identify critical factors related to patient falls through the application of data mining to available data through a hospital information system.
(76)	2021	Proactive	This study evaluated whether natural language processing of psychotherapy note text provides additional accuracy over and above currently used suicide prediction models.
(62)	2021	Reactive	The presented Incident report classification framework aimed to improve the identification of the fall severity level mainly by incorporating structured features and leveraging resampling methods.
(42)	2019	Reactive	This work uses text mining to analyze fall incident reports, automatically grouping them based on semantic content for retrospective study.
(43)	2019	Reactive	The project developed a predictive model for Roche/Genentech to oversee adverse event reporting across program, study, site, and patient levels, integrating advanced analytics with traditional quality assurance approaches.
(75)	2013	Proactive	This paper aims to provide to the technology decision makers in healthcare (Health Management, Clinical Engineering and Prevention and Protection Service) a decision support system for analyzing the safety level associated to the use of technology for both patients and personnel.
(74)	2018	Reactive	The paper presents a knowledge discovery framework, the Safer Dx Trigger Tools Framework, that enables health systems to develop and implement e-trigger tools to identify and measure diagnostic errors using comprehensive electronic health record data.
(44)	2020	Reactive	The research aimed to establish a method to extract incident candidates from clinical notes in order to detect non-reported severe incidents. In addition, we implemented a reporting system that presents incident candidates extracted by using the pro- posed method.
(45)	2012	Reactive	The paper explores the feasibility of using statistical text classification to automatically detect extreme-risk events in clinical incident reports.
(46)	2020	Reactive	The main objective of the study is the construction of a model that could determine timely, on a near real time, if the patient readmission within 30 days was associated with a hospital acquired adverse event that occurred in the previous admission (response variable).
(63)	2019	Proactive	the paper developed a model for predicting falls using interpretable machine learning and integrating the model into the electronic medical record system to perform nursing interventions for each risk factor.
(77)	2021	Proactive	This research assesses the model by building a machine learning-based algorithm and altering network settings, then confirms the suggested technique using actual disinfectant supply center data.
(64)	2023	Proactive	The objective is to compare the performance of machine-learning models with the Medication Fall Risk Score in predicting fall risk related to prescription medications.
(73)	2023	Reactive	This paper assesses how staff experience impacts reported error rates in patient and staff safety using machine learning to identify key dimensions and variables influencing safety outcomes.
(71)	2021	Proactive	We used machine learning to develop an advanced tool for early assessment of pressure injury risk, leveraging extensive clinical data routinely recorded in electronic health records.
(47)	2018	Reactive	The aims of this study are two-fold. Firstly, to assess the technical feasibility of an application for reporting incidents that combines a conversational interface with speech recognition software. Secondly, to undertake a pilot study of its usability for clinical contexts.
(72)	2023	Proactive	This paper comprehensively reviews artificial intelligence and Decision Support System applications for hospital-acquired pressure injuries prediction using Electronic Health Records, including a systematic literature review and bibliometric analysis.
(65)	2019	Reactive	The purpose of this study is to build a practical system useful to predict the severity level of in-hospital falls.
(66)	2019	Proactive	The objective of this study is to develop a general predictive model for severity of falls among patient populations, using an advanced machine learning method multi-view ensemble leaning to efficiently exploit the multidimensional patient data.
(48)	2020	Reactive	The study evaluates the utility of semantic feature representation for automated identification incident reports by type and severity.
(49)	2019	Reactive	The aim is to develop a single classifier by combining word embedding with a Convolutional Neural Networks and to evaluate its feasibility to identify multiple types of incident reports and severity levels.
(50)	2017	Reactive	The paper evaluates the feasibility of using multilabel classification to automate the identification of two labels or two incident types per report.
(55)	2020	Proactive	The aim of this study was to develop a medication-rights detection system to classify medication incidents using the real-world incident reports collected by the Hong Kong Hospital Authority.
(51)	2019	Reactive	The aim is to perform a systematic literature review and narrative synthesis to describe and evaluate natural language processing methods for classification of incident reports and adverse events in healthcare.
(56)	2023	Proactive	This study aimed to gather pharmacists feedback in a focus group setting to help inform the initial design of the user interface and iterative designs of the AI prototype.
(52)	2018	Proactive	In the paper a pipeline is proposed to help clinicians deal with the accumulated reports, extract valuable information and generate feedback from the reports

### 3.2 Publication period

**Figure 2:**
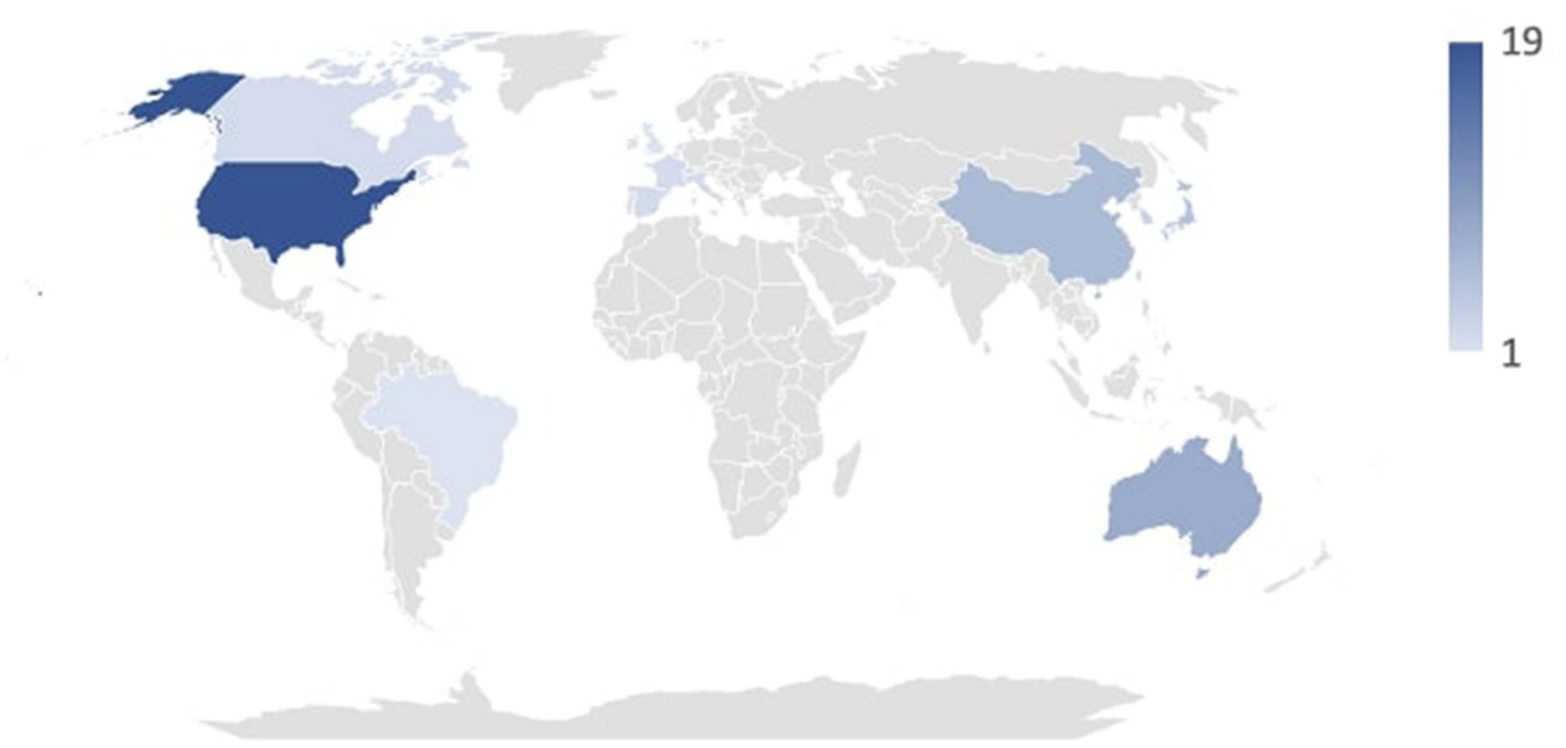
Geographical distribution of publications.

The articles under review were published between 2007 and 2024. As expected, the number of publications has seen a steady increase in recent years due to growing interest, particularly in media coverage, and the development of artificial intelligence systems. Specifically, from 2019 to 2024, 36 of the analyzed studies were produced, compared to 16 from 2007 to 2008. As depicted in Figure 2, the countries where the analyzed studies originated include Israel, Denmark, Netherlands, Lebanon, Brazil, United Kingdom, Switzerland, Canada, France, Italy, Spain, United Arab Emirates, South Korea, Taiwan, Japan, China, Australia, and the United States. Figure 2 shows the duration in years of the studies that provided this type of information.

### 3.3 Results for single topics

In this systematic review, it emerged that the most frequently discussed topics in the scientific literature related to risk management are related to the detection of adverse events, followed by the risk of falls, and then the development of pressure ulcers.

#### 3.3.1 Detection of adverse events

In the study conducted by Barmaz Y and Ménard T (30), a hierarchical Bayesian model was employed to estimate reporting rates at clinical sites and assess the risk of under-reporting based on anonymized public clinical trial data from Project Data Sphere. This model infers reporting behavior from patient data, enabling the detection of anomalies across clinical sites. This system has proven useful by reducing the need for audits and enhancing clinical quality assurance activities related to safety reporting in clinical trials. Bates et al. (31) conducted a scoping review evaluating the role of AI in improving patient safety through the interpretation of data collected from vital signs monitoring systems, wearables, and pressure sensors. The evidence gathered recognized significant potential in this approach, though continuous efforts are required for implementing these systems in healthcare organizations. Benin et al. (32) developed an electronic system for processing medical event reports to enhance patient safety. This system improved care safety outcomes by categorizing the same event into multiple error categories based on logical correspondences, unlike manual approaches where each error type corresponds to a single category. Elizabeth M. Borycki's work (33) addressed incident reporting related to adverse events induced by healthcare technologies, assessing the associated advantages and disadvantages. The study concluded that this experimental approach is promising. Chen H et al. (34) evaluated the effectiveness of various machine learning-based automatic tools for adverse event classification, proposing an interface integrated with this system. The results highlighted the potential of such a system to achieve efficient and reliable report classification processes. Similarly, S. Fodeh et al. (35) proposed an automatic classification model for adverse events, combining feature detection system operations with a machine learning classifier. This model proved particularly useful for two adverse event categories: patient identification errors and weight-related issues. In contrast, Allan Fong et al. (36) advocated for the use of natural language processing (NLP) in identifying four categories of errors: Pharmacy Delivery Delay, Pharmacy Dispensing Error, Prescriber Error, and Pyxis Discrepancy. The study demonstrated that the tool's accuracy can help reduce the workload of hospital safety committees. Katsuhide Fujita et al. (37) applied NLP in incident reporting to analyze incident report texts, reinterpreting structured incident information and improving incident-related cause management. The article highlighted the tool's effectiveness, particularly for issues related to patient falls and medication management. Gerdes and Hardahl (38) tested an NLP system for reviewing clinical records to identify adverse events. The encouraging results suggest considering the systematic introduction of such automatic monitoring systems. Gupta et al. (39) proposed an automatic clinical incident classification system testing four different algorithms. Among these, the multinomial naive Bayes algorithm demonstrated particular efficiency, requiring a well-structured training phase. In another study, Gupta J et al. (40) introduced an incident reporting system based on the C4.5 decision tree algorithm and random forest, using a taxonomy from a generic system and one proposed by the WHO. The study demonstrated the superiority of the random forest algorithm and introduced a modification to the WHO taxonomy by adding another adverse event class. Hendrickx et al. (41) applied text mining techniques to highlight patient safety issues, indicating that these systems can be useful for prioritizing safety concerns and automatically classifying event severity. Liu et al. (42) proposed a text mining system for retrospective analysis of patient fall reports, reporting highly encouraging results regarding its application. Ménard et al. (43) proposed an under-reporting detection system for adverse events using a machine learning approach. Positive results from clinical trials of this approach led to the extension of this adverse event detection system to all future Roche/Genentech studies. Okamoto et al. (44) employed a machine learning system to detect unreported errors in medical records, identifying 121 incidents, with 34 subsequently selected as serious errors. In their work, Ong et al. (45) explored using Naïve Bayes and SVM text classifiers to detect extreme-risk events in clinical reports from Australian hospitals. The classifiers were evaluated on their accuracy, precision, recall, F-measure, and AUC, showing feasibility for automatic detection of high-risk incidents. Implementing a fall risk prediction tool resulted in a reduction in patient falls and an increase in risk-targeted nursing interventions in intervention units, although there was no significant difference in fall injury rates compared to control units. Saab et al. (46) proposed a machine learning model for predicting adverse events responsible for hospital readmission, aiming to reduce associated costs. The achieved accuracy levels were consistent with previous studies, highlighting the real-time feedback advantage of the tested system. Sun et al. (47) proposed an incident reporting system combining a conversational interface with speech recognition software, concluding that socio-technical issues currently preclude its implementation. Wang et al. (48) evaluated the feasibility of using the Unified Medical Language System (UMLS) for automatically identifying patient safety incident reports by type and severity, showing its superiority over bag-of-words classifiers. In another study by Wang et al. (49), neural networks were used to assess the severity and gravity of adverse event reports. In a third study by Wang et al. (50), a multi-label incident classification system was structured for multiple incident types in individual reports. While not broadly applicable, this method proved useful in multi-label classification using a support vector machine algorithm. In a systematic review by Young et al. (51), NLP was investigated for free-text recognition in incident reporting. The review concluded that NLP can yield significant information from unstructured data in the specific domain of incident and adverse event classification, potentially enhancing adverse event learning in healthcare. Zhou et al. (52) proposed and tested an automated system for analyzing medication dispensing error reports based on machine learning algorithms. The study developed three different classifiers based on two algorithms (support vector machine and random forest), capable of identifying event causes and reorganizing them based on similarities.

#### 3.3.2 Medication-related error

In a study by Corny et al. (53), a hybrid clinical decision support system was tested to reduce errors in the medication prescribing phase. Implementing this system demonstrated higher accuracy compared to existing techniques, intercepting 74% of all prescription orders requiring pharmacist intervention, with a precision of 74%. King et al. (54) used machine learning models to predict medication ordering errors and identify contributing factors. Decision trees using gradient boost achieved the highest AUROC (0.7968) and AUPRC (0.0647) among all models, showing promise for error surveillance, patient safety improvement, and targeted clinical review. Wong et al. (55) proposed a wound dressing rights detection system using NLP and deep neural networks. This system automated the identification of dressing incidents, highlighting the potential of deep learning for exploring textual reports on dressing incidents. Zheng et al. (56) focused on medication dispensing errors, reporting the development of an AI system through collaboration with pharmacists. They improved various features such as the interpretability of AI systems by adding gradual check marks, probability scores, and details on medications confused by the AI model. They also emphasized the need to build a simple and accessible system.

#### 3.3.3 Patient fall risk

Stein et al. (57) evaluated the impact of a fall risk prediction system, assessing its outcomes in terms of patient outcomes and nurse feedback. The results highlighted a slight immediate reduction in the number of falls without consistent long-term effects, but the tool demonstrated intrinsic utility. Cho et al. (58) evaluated the usability of a predictive algorithm for detecting individual fall risk factors. Although a reduction in fall rates was observed, particularly in those over 65 years old, the intervention was not associated with a significant reduction in this rate. Huang et al. (59) employed a machine learning approach to study a 14-month fall event database aimed at developing a predictive fall risk system. This approach demonstrated particular accuracy and is used daily in one of Taiwan's medical centers. Ladios-Martin et al. (60) developed a machine learning tool for fall risk prediction through the evaluation of a series of variables in a retrospective cohort. The Two-Class Bayes Point Machine algorithm was chosen, showing a reduction in fall events compared to the control group. Lee et al. (61) used a different approach to falls, employing data mining on hospital information system data. An artificial neural network was used to develop a predictive model that demonstrated high predictivity with a higher ROC compared to a logistic regression model. Liu et al. (62) proposed a system aimed at improving and automating severity classification models of incidents. The tool proved useful in identifying and classifying fall events, with the top two algorithms being random forest and random oversampling. Shim et al. (63) developed and validated a machine learning model for fall prediction that is integrable into an electronic medical record system. This system, whose effectiveness was confirmed during the study, was subsequently officially integrated into the clinical record system. Silva et al. (64) proposed a machine learning model based on the Naive Bayes algorithm for developing a predictive tool for patient fall risk related to prescribed drug therapy. The Naive Bayes algorithm demonstrated superior values compared to other algorithms, particularly with an AUC of 0.678, sensitivity of 0.546, and specificity of 0.744. Wang et al. (65) proposed a tool to predict the severity of damage following a patient fall. Several machine learning algorithms were used, with the random forest algorithm proving the best with an accuracy of 0.844 and precision of 0.839. Therefore, an online severity prediction system was built using the RF algorithm and Flask package. By leveraging this predictive system, healthcare facilities can enhance patient safety and better allocate limited resources. Wang et al. (66) proposed a model to evaluate the predictability of fall events among hospitalized patients through a retrospective cohort study. A predictive classifier developed using multi-view ensemble learning with missing values demonstrated superior predictive power compared to random forest and support vector machine, two other comparison algorithms.

#### 3.3.4 Pressure injury

Barghouthi et al. (67) conducted a systematic review of prediction models for the development of pressure ulcers. The study highlighted that the most commonly employed algorithm is logistic regression. However, it also noted that none of the reviewed studies successfully used the pressure ulcer prediction model in real-world settings. Borlawsky and Hripcsak (68) proposed a similar model based on the C4.5 decision tree induction algorithm. Results showed limited application of this naive classification algorithm to automate the assessment of pressure ulcer risk. Do et al. (69) assessed the impact of an electronic predictive tool on fall risk using EHR data compared to a standard assessment tool. Conducted over 2 years in 12 nursing units, the primary outcome measured was the rate of patient falls, with secondary outcomes including injury rates and nursing interventions. The most accurate model achieved a 99.7% area under the receiver operating characteristic curve, with ten-fold cross-validation ensuring generalizability. Random forest and decision tree models had the highest prediction accuracy rates at 98%, consistent in the validation cohort. Ladios-Martin et al. (70) proposed another predictive model for the risk of developing pressure ulcers using a logistic regression algorithm. The model demonstrated a sensitivity of 0.90, specificity of 0.74, and an area under the curve of 0.89. The model performed well 1 year later in a real-world setting. Song et al. (71) employed a random forest-type predictive algorithm applied to a case study of hospital-acquired and non-hospital-acquired pressure ulcers, showing AUCs of 0.92 and 0.94 in two test sets. The study concluded that the tool could also be employed in real-world settings. Toffaha et al. (72) reviewed the literature on the prediction of pressure ulcer development, highlighting the existence of numerous predictive models, none of which have been applied in real healthcare settings but were rather trained on previous cases.

#### 3.3.5 Other areas of clinical risk management

Simsekler et al. (73) employed three different machine learning algorithms to identify potential associations between organizational factors and errors affecting patient and staff safety. The results suggested that “health and wellbeing” is the main theme influencing patient and staff safety errors, with “workplace stress” being the most important factor associated with adverse outcomes for both patients and staff. Murphy et al. (74) proposed a system known as Safer Dx Trigger Tools, capable of identifying real-time and retrospective errors in the care pathway through analysis of electronic clinical data. The study concluded with the potential future application of this type of tool in daily hospital practice. Miniati et al. (75) provided a decision support system for analyzing the safety level associated with the use of technologies for both patients and staff. The experimental tool proved useful in predicting outcomes in specific scenarios, with the authors concluding that this could be extended to other areas. Levis et al. (76) analyzed the suicide risk factor through retrospective analysis of psychiatric notes using a predictive model based on NLP. Specifically, an 8% increase in predictability was observed in the 12-month study cohort compared to more advanced available methods. Hui Jun Si et al. (77) proposed a risk management model related to the disinfection process of hospital environments using AI systems. Using a k-nearest neighbor algorithm, the results highlighted that levels of job satisfaction and work standardization achieved by nursing staff managed by an AI algorithm were significantly higher than those achieved by nurses working in traditionally managed disinfection centers. Huang et al. (78) proposed a system known as Artificial Intelligence Clinical Assistant Decision Support System (AI-CDSS) for preventing thromboembolic events; however, the tool was found to be ineffective. Choundhury et al. (79) conducted a literature review on the role of AI in ensuring patient safety, focusing on subcategories such as clinical alarms, clinical reports, and medication safety issues. Several software analyzed in this study have been designed and developed with features that can be considered medical devices, however, according to the literature reviewed, none of them have reached an official approval stage according to the EU MDR 2017/745 regulation or the US FDA. According to EU MDR 2017/745, among other aspects, software can be considered a medical device if it is intended to provide information for diagnostic and therapeutic purposes, as well as to help prevent, monitor, diagnose or even treat disease or injury (80). Based on this, the model developed by Corny et al. (53), which identifies prescriptions with a high risk of error, could fall into this category, as could the one proposed by Ladios-Martin et al. (6) for fall prevention. User acceptance and specific training are central aspects for the successful implementation of artificial intelligence (AI) systems in clinical settings. Barriers such as resistance to change, technological complexity, and lack of specific expertise can be overcome with targeted strategies such as user-centered design and dedicated training programs. Many studies included in the review highlight the importance of involving end users (physicians, nurses, pharmacists) early in development to ensure that systems meet their operational needs. Targeted training, often supported by pilot testing and simulations, has proven crucial in familiarizing users with new technologies and improving their confidence in daily use. For example, Sun et al. designed a speech recognition-based reporting system, the use of which was tested through a pilot project. The feedback highlighted the need for more detailed instructions to overcome the socio-technical difficulties encountered (47). Silva et al. developed a predictive model for fall risk and accompanied its implementation with specific training sessions. Users evaluated the approach positively, emphasizing the usefulness of ongoing support (64). Similarly, Huang et al. highlighted how practical training sessions improved the adoption of a predictive system for falls risk, facilitating the integration of the software into clinical practice and gathering suggestions for further technical improvements (59). Zheng et al. developed a system to prevent medication dispensing errors using focus groups with pharmacists. This approach allowed them to iterate on the interface and instructions for use, significantly improving end-user satisfaction (56). According to the review, most studies did not highlight significant issues with AI, such as the lack of standards and evaluation metrics. Further research and involvement of FDA and NIST are needed to create standards that ensure patient safety.

## 4 Discussion

The reviewed studies primarily focus on incident reporting in healthcare, with two prominent approaches: automatic incident classification systems and event detection through healthcare documentation analysis. Machine learning algorithms have proven effective in automating incident classification, enhancing accuracy through past case training. Natural language processing and text mining techniques have enabled automated adverse event detection and anomaly identification in clinical data, improving care quality and reducing manual audits. Continuous implementation and system refinement are crucial for maximizing these benefits and addressing socio-technical challenges in healthcare settings. In managing medication errors, AI and machine learning have shown promise in decision support for prescription accuracy and error prevention during medication ordering. Hybrid clinical decision support systems and gradient boosting decision trees demonstrate significant accuracy in intercepting prescription errors. Deep learning techniques improve medication incident identification, emphasizing collaboration with pharmacists for system interpretability and usability in clinical practice. Regarding falls management, AI applications focus on predictive models for fall risk and severity classification systems. While predictive algorithms enhance risk assessment, their impact on reducing falls varies across age groups and implementation settings within electronic health records. Ongoing refinement is necessary to optimize predictive accuracy and practical integration into clinical workflows. Studies on predictive models for pressure ulcer development reveal varied efficacy, with machine learning algorithms like random forest showing promising predictive capability. However, the application of these models in real healthcare environments requires further validation and standardization to ensure practical clinical utility. AI and machine learning also play pivotal roles in enhancing patient and healthcare staff safety. They identify organizational factors influencing safety outcomes, support real-time error detection through tools like Safer Dx Trigger Tools, and improve predictive accuracy for technology-related risks and suicide risk. Despite successes, challenges remain, including the need for standardized evaluation metrics and regulatory oversight to ensure the efficacy and safety of AI applications in patient care. A crucial issue remains the proper and safe implementation of AI in clinical risk management practices. First, it is crucial to assess the specific needs of the clinical setting by going out and identifying all the areas where AI can provide the greatest positive impact, such as adverse event detection, falls prevention, or medication error management. This type of analysis should, in any case, involve end users so that the system is designed and designed based on their operational needs. This should be followed by a controlled pilot phase to test the technology in a protected environment to highlight possible problems related to its use; at this juncture, safety measures such as automated monitoring and audit systems should be implemented to reduce bias and errors (81). At a second stage, user education with training programs to understand the technical operation of the system but also its limitations should be crucial. Once the system is validated, its large-scale implementation should be accompanied by continuous monitoring with periodic audits and user reporting systems. Finally, the AI system should be designed to work in perfect synergy with existing tools such as hospital information systems and electronic health records. Such a holistic approach could not only improve the safety and quality of care but could also optimize the allocation of healthcare resources.

## 5 Conclusions

The reviewed studies demonstrate that artificial intelligence (AI) and machine learning (ML) systems are transforming healthcare safety across various domains, including incident management, medication prescription, and fall prevention. Predictive algorithms and ML models have significantly improved the identification and handling of adverse events, reducing reliance on manual audits and enhancing reporting accuracy. Despite these advancements, the practical application of AI in real healthcare settings remains limited and requires ongoing refinement. Future efforts aim to enhance these systems by integrating feedback from healthcare professionals and optimizing their integration with electronic health records. Establishing uniform standards and evaluation metrics is critical to ensuring the effectiveness and safety of AI-driven solutions. Collaboration with regulatory bodies is essential to develop guidelines that support the safe and efficient use of AI technologies in everyday clinical practice. These advancements are expected to not only enhance care quality but also facilitate more effective management of healthcare resources.

## Data Availability

The original contributions presented in the study are included in the article/supplementary material, further inquiries can be directed to the corresponding author.
